# Dual Path Lock-In System for Elimination of Residual Amplitude Modulation and SNR Enhancement in Photoacoustic Spectroscopy

**DOI:** 10.3390/s18124255

**Published:** 2018-12-04

**Authors:** Qinduan Zhang, Jun Chang, Zhenhua Cong, Zongliang Wang, Fupeng Wang

**Affiliations:** 1 School of Information Science and Engineering and Shandong Provincial Key Laboratory of Laser Technology and Application, Shandong University, Jinan 250100, China; zhang_qinduan@163.com (Q.Z.); congzhenhua@sdu.edu.cn (Z.C.); wfp1990@uw.edu (F.W.); 2 School of Physics Science and Information Technology and Shandong Key Laboratory of Optical Communication Science and Technology, Liaocheng University, Liaocheng 252059, China; wangzongliang@lcu.edu.cn

**Keywords:** dual path lock-in, RAM, photoacoustic spectroscopy

## Abstract

A technique for elimination of residual amplitude modulation (ERAM) in photoacoustic spectroscopy based on dual path lock-in was proposed and experimentally demonstrated. There are two lock-in amplifiers, one is for gas concentration demodulation and another for residual amplitude modulation (RAM) measurement by tuning the reference signal in different phases, and then a dual path lock-in technique based on subtraction is applied to RAM removal, improving the second harmonic profile significantly. In this system, the signal to noise ratio (SNR) increases about two times based on our dual path lock-in technique compared to one distributed feedback laser diode (DFB-LD). The system achieved a good linear response (R-square = 0.99887) in a concentration range from 100 ppmv to 2400 ppmv and a minimum detection limit (MDL) of 1.47 ppmv.

## 1. Introduction

The photoacoustic spectroscopy (PAS) technique is one of the most sensitive techniques in trace gas detection. In recent years, PAS has been widely presented as a reliable technique to detect the concentration of gases such as C_2_H_2_ [1], H_2_O [2], CO_2_ [3], CH_4_ [4] and so on. The amplitude modulation technique [5] and wavelength modulation technique [6] are commonly used in PAS gas sensors. Amplitude modulation technique-based PAS is realized by amplitude modulation through a chopper or an acousto-optic modulator (AOM), and the detection frequency of the lock-in amplifier is equal to the modulation frequency. Thus, the sensitivity of trace gas detection may be limited by periodic heating of the cell walls. The wavelength modulation technique is implemented by applying a modulation signal (modulation frequency = half of the PAS cell resonance frequency *f*_0_) to the diode laser current and detecting the PAS cell response at *f*_0_ using a lock-in amplifier. It is well known that PAS sensor with wavelength modulation technique requires an accurate second harmonic signal waveforms. In reality, second harmonic signal waveforms will be distorted, which is referred to, widely, as the residual amplitude modulation (RAM), when the diode laser wavelength (or frequency) is modulated through its injection current. Therefore, the recovery of the second harmonic signal waveforms have attracted growing interest.

The origin of RAM and its distorting effects on wavelength modulation spectroscopy, especially on various harmonic signals, have been studied in Kluczynsk’s report [7]. Recently, some techniques have been reported for RAM effect suppression. Wei et al. [8] presented a modulation index adjustment technique for second harmonic signal waveforms recovery. The effectiveness of this method was verified by experiments and theoretical calculations. Chakraborty et al. [9] reported a method to suppress RAM using an optical fiber delay line and it can lead to improvements in sensitivity for 1st harmonic detection schemes. Tommasi et al. [10] showed that a drastic reduction of RAM effect is possible using an intensity-control feedback loop, based on an acoustooptic modulator. Zhu et al. [11] introduced a continuously wavelength-tunable light source with constant power output, by which the spectral distortion of second harmonic signal caused by RAM was greatly improved.

In the PAS system, the PAS signal is directly proportional to the incident laser power. Therefore, many researchers are devoted to improving the incident power in PAS systems. Wang et al. [12] proposed the concept of fiber-ring laser intracavity photoacoustic spectroscopy. The PAS gas cell was placed inside the fiber-ring laser to fully utilize the intracavity laser power, and this achieved a 390 ppbv minimum detection limit (MDL). Ma et al. [13] employed an erbium-doped fiber amplifier (EDFA) to amplify DFB-LD with a central wavelength of 1.53 µm, which was used as the excitation source, and a MDL of 33.2 ppbv was estimated for C_2_H_2_ detection. Liu et al. [14] demonstrated a method to enhance the PA signal with a right angle prism. Compared to the traditional method without the right angle prism, the system signal to noise ratio (SNR) has been improved from 1113 to 1928.

In this paper, we propose a dual path lock-in technique based on subtraction that is applied to eliminate residual amplitude modulation and SNR enhancement. A low frequency photoacoustic cell is used in this system. The application of lock-in amplifier in low frequency experiment was verified by Macias-Bobadilla et al. [15]. In this system, the original second harmonics and the RAM components from the first harmonics and third harmonics are measured separately in the first part, which is later used to recover the absolute absorption signals from original second harmonics based on subtraction.

## 2. Theory Analysis

### 2.1. ERAM Theory

According to the Beer-Lambert law [16], when a probe laser passes through the gas medium and is absorbed by gas molecules, the transmitted laser power can be expressed by Equation (1):

(1) $$I_{OUT}=I_{IN}\cdot \exp\left[-\phi_{L}(\nu )SPC\cdot L\right]$$

*I_IN_* stands for the incident laser power and *I_OUT_* stands for the transmitted laser power. *S* is the absorption line intensity, *φ_J_*(*ν*) is the linear function of gas absorption spectra, *P* is the gas cell pressure, *C* is the gas concentration, *L* is the absorption path-length. When the output power of the laser is modulated periodically, the output optical frequency *ν_C_* and incident laser power *I_IN_* can be expressed as Equations (2) and (3):

(2) $$\nu_{c}=\nu +\Delta \nu \cos(\omega t-\Delta \varphi )$$

(3) $$I_{IN}=I(\nu_{c})+\Delta I\cos(\omega t)$$

where *ν* is the modulation center frequency and Δ*ν* is frequency modulation amplitude. *I*(*ν_C_*) is the laser power of the corresponding modulation center frequency, Δ*I* is the intensity modulation amplitude, *ω* is the modulation angle frequency, *ω* = 2*πf*, *f* is the modulation frequency (half of the resonant frequency of the PAS cell), Δ*ϕ* is the phase difference between the laser output power and the optical frequency (output wavelength of the laser) [17].

According to Equations (1)–(3), the output laser power *I_OUT_* can be expressed as:

(4) $$\begin{array}{c} I_{OUT}=\left(I(\nu_{c})+\Delta I\cos(\omega t)\;\right)\cdot \exp\left[-\phi_{L}(\nu )SPC\cdot L\right]\\ =\left(I(\nu_{c})+\Delta I\cos(\omega t)\;\right)\cdot \exp\left[-\alpha \left(\nu \right)\cdot L\right] \end{array}$$

For the gas absorption line at room temperature and pressure, we consider the case of Lorentzian profile, the absorption coefficient *α*(*ν*) is defined by:

(5) $$\alpha (\nu )=\frac{SPC}{2}\frac{\Delta \nu_{L}}{(\nu_{c}-\nu_{0}+\Delta \nu \cos(\omega t-\Delta \varphi ))+\left(\Delta \nu_{L}/2\right)}$$

Δ*ν_L_* is the full width at half maximum (FWHM) of the absorption line. Making *γ* = Δ*ν_L_*/2, *X* = (*ν_L_* − *ν*_0_)*γ*, *m* = Δ*ν*/*γ*, *m* is the optical frequency modulation factor. Equation (5) can be represented as:

(6) $$\alpha (\nu )=\frac{SPC}{\pi \gamma }\frac{1}{{\left(X+m\cos(\omega t-\Delta \varphi )\right)}^{2}+1}=a_{0}\frac{1}{{\left(X+m\cos(\omega t-\Delta \varphi )\right)}^{2}+1}$$

The absorption coefficient *α*(*ν*) can be deduced by using the Fourier expansion method as:

(7) $$\alpha (\nu )=a_{0}\left[H_{0}+\sum_{n=1}^{\infty }H_{n}\cos\left(n\omega t-n\Delta \varphi \right)\right]$$

where *H*_0_ and *H_n_* is the Fourier expansion coefficients.

According to Equations (4) and (7), the harmonic signal can be expressed as:

(8) $$\begin{array}{l} I_{2H}=-\frac{1}{2}\Delta Ia_{0}H_{1}\cos(2\omega t-\Delta \varphi )-I\left(\nu_{c}\right)a_{0}H_{2}\cos(2\omega t-2\Delta \varphi )\\ -\frac{1}{2}\Delta Ia_{0}H_{3}\cos(2\omega t-3\Delta \varphi ) \end{array}$$

In second harmonic signal-based gas concentration detection, a reference signal with a frequency of 2*ω* is used to extract the second harmonic signals. Two reference signals with different phases *θ* and *ψ* are generated to detect the second harmonic signals, respectively, from lock-in amplifier 1 and lock-in amplifier 2 as Equations (9) and (10), in which item *q*_1_ is the principal second harmonic component and item *q*_2_ is RAM coming from first harmonics and third harmonics. *B* is a constant.

(9) $$U_{1}=\underset{q_{1}}{\underset{︸}{-\frac{1}{2}a_{0}H_{2}B\cos\left(\theta +2\Delta \varphi \right)}}\underset{q_{2}}{\underset{︸}{-\frac{1}{4}a_{0}H_{1}B\cos\left(\theta +\Delta \varphi \right)-\frac{1}{4}a_{0}H_{3}B\cos\left(\theta +3\Delta \varphi \right)}}$$

(10) $$U_{2}=\underset{q_{1}}{\underset{︸}{-\frac{1}{2}a_{0}H_{2}B\cos\left(\psi +2\Delta \varphi \right)}}\underset{q_{2}}{\underset{︸}{-\frac{1}{4}a_{0}H_{1}B\cos\left(\psi +\Delta \varphi \right)-\frac{1}{4}a_{0}H_{3}B\cos\left(\psi +3\Delta \varphi \right)}}$$

When tuning *θ* to make *θ* + 2Δ*ϕ* = 0, item *q*_1_ of *U*_1_ achieves a maximum value. Tuning *ψ* to make *ψ* + 2Δ*ϕ* = *π*/2. Only RAM item *q*_2_ coming from first harmonics and third harmonics is left, item *q*_1_ of *U*_2_ becomes zero. Then, signal *U*_1_ minus signal *U*_2_ gives:

(11) $$U_{OUT}=\kappa \left(U_{1}|\theta +2\Delta \varphi =0\right)-\varepsilon \cdot \left(U_{2}|\psi +2\Delta \varphi =\pi /2\right)$$

*κ* and *ε* are the adjustment factors.

Thus, the RAM-caused distortion can be eliminated effectively by adjusting the *κ* and *ε* factors. Furthermore, the second harmonic signal amplitude will not be reduced in this method.

### 2.2. SNR Enhancement Theory

In the PAS system, the detected photoacoustic signal *S* is related to the gas absorption coefficient *α*(*ν*) and the incident laser power *I_IN_*:

(12) $$S\propto C\cdot \alpha \left(\nu \right)I_{IN}Q/f_{0}$$

where *Q* and *f*_0_ are the quality factor and resonant frequency of the acoustic resonator, respectively. The linear relationship between the PAS signal amplitude and the incident laser power provides the attractive feature that the sensitivity of PAS-based sensors benefits from high-power laser [13].

## 3. Presentation of Experimental Setup

**Figure 1:**
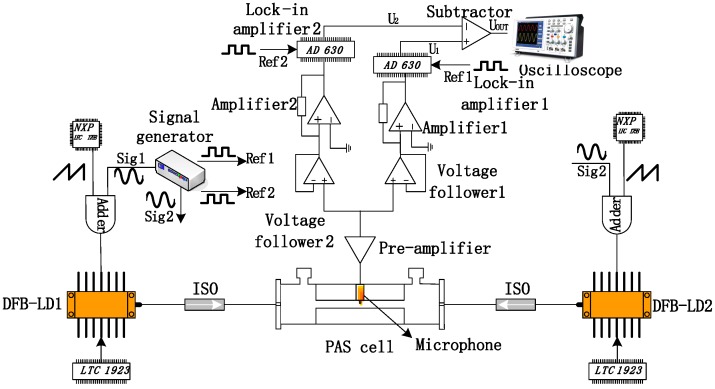
Schematic of the dual path lock-in system.

**Figure 2:**
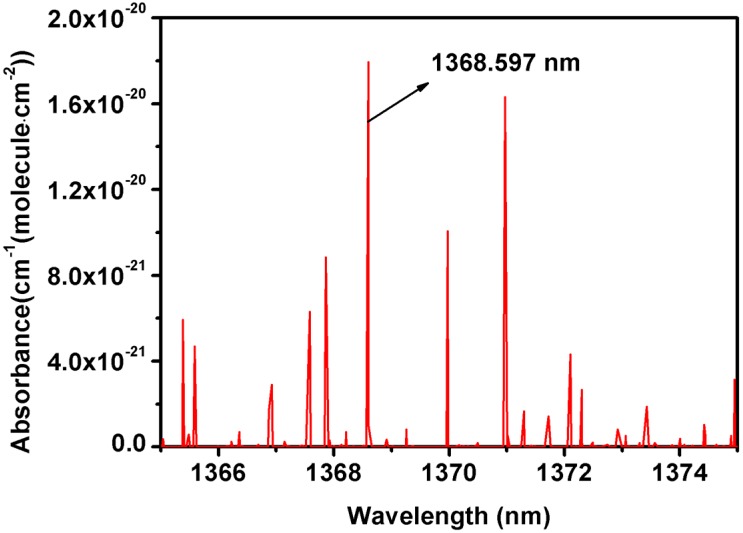
H_2_O absorption lines in the range of 1365–1375 nm.

The schematic diagram of dual path lock-in system in photoacoustic spectroscopy is shown in Figure 1. Water vapor has a strong absorption line at 1368.597 nm (7306.752 cm^−1^) according to HITRAN 2012 [18] (see Figure 2), so two single-mode DFB-LDs (DFB-1368-F-N; Wuhan 69 Sensor Technology, Wuhan, China) are used as the laser source, with an output wavelength of approximately 1368 nm. Two micro temperature controlling chips (LTC 1923, Linear Technology, Milpitas, CA, USA) are utilized to control the temperature of DFB-LDs. The sawtooth wave signals are generated by the chip LPC1758 to scan the absorption line with a sweep range of about 300 pm. Simultaneously, the high-frequency sinusoidal wave signals (Sig1 and Sig2) are generated by a function signal generator (FY2300A, Feel Tech, Zhengzhou, China). The adders add the two signals as laser drive signal. The DFB-LDs are connected to the G-lens fiber collimators, which can be directly inserted inside the PAS cell. 

Two G-lens are respectively placed at the two ends of the custom made transmission photoacoustic cell. The transmission photoacoustic cell is characterized to show a resonant frequency of 5.3 kHz. The isolators (ISO) are used to avoid laser entering the DFB-LDs. A miniature electret microphone (EK-3024, Knowles, Itasca, IL, USA) mount at the center of the transmission photoacoustic cell is used to detected the excited PAS signal. The PAS signal is amplified by a pre-amplifier chip (CA3140E, Intersil, Milpitas, CA, USA). A custom made dual lock-in circuit based on two AD630 chips [19] is used to extract the harmonic signals. The reference signals Ref1 and Ref2 of lock-in circuit are provided by the function signal generator. The amplifier 1 and amplifier 2 are used to adjust the κ and ε factor as discussed in Equation (11). The two signals output from the lock-in amplifiers are subtracted by a subtractor to eliminate the residual amplitude modulation.

## 4. Experimental Verification

### 4.1. Elimination of Residual Amplitude Modulation

**Figure 3:**
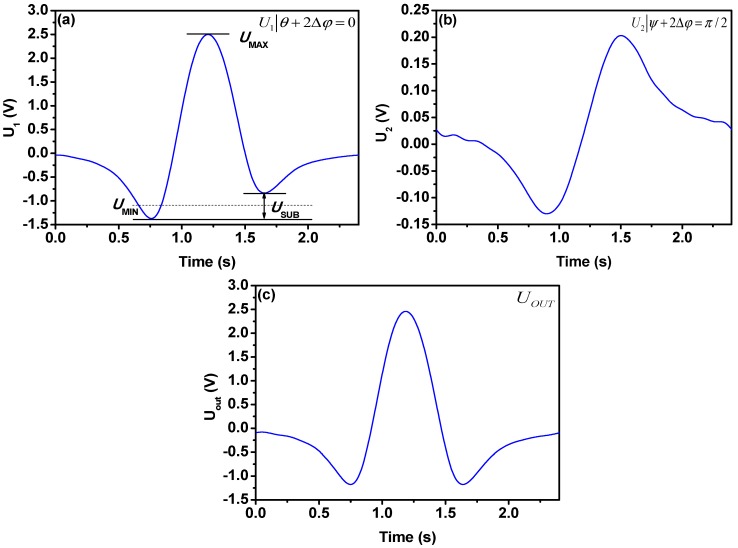
(**a**) Second harmonic signal output from lock-in amplifier 1, (**b**) The RAM components output from lock-in amplifier 2, (**c**) The second harmonic signals after applying the dual path lock-in technique, the RAM effects are totally eliminated.

A home-made dew-point generator was used to produce 2000 ppmv water vapor samples. Precision of the dew-point generator is within 1 ppmv in the range of 80–2500 ppmv. All the measurements were performed at the pressure of 1 bar and room temperature (24 °C). The two DFB-LDs worked simultaneously. By adjusting the micro temperature control circuit, the gas absorption peak was adjusted to the center of the sawtooth wave. The waveforms acquired in a single cycle are shown in Figure 3a–c.

Adjusting the phase *θ* of reference signal (Ref. 1) to make *θ* + 2Δ*ϕ* = 0, the original second harmonic signal was measured as in Figure 3a. When tuning the phase *ψ* of reference signal (Ref. 2) to make *ψ* + 2Δ*ϕ* = *π*/2, the RAM coming from first and third harmonics was measured as seen in Figure 3b. Adjustment of *κ* and *ε* is realized by tuning the amplifier 1 and amplifier 2, and the second harmonic signal without RAM is plotted in Figure 3c.

Usually, an asymmetry factor (AF) [11] is used to evaluate the degree of spectral asymmetry caused by RAM effect, and it is expressed as:

(13) $$AF=\frac{U_{SUB}}{U_{MAX}-U_{MIN}}$$

where *U_SUB_* is the difference of the two minima of the second harmonic signal, and *U_MAX_* is maximum value of the second harmonic signal, *U_MIN_* represents the mean value of the two minima. As can be seen from Figure 3a, the AF value of the original second harmonic signal output by the lock-in amplifier is about 0.213. When dual path lock-in technique was applied, AF reduces to 0, which can be seen from Figure 3c.

### 4.2. SNR Enhancement

**Figure 4:**
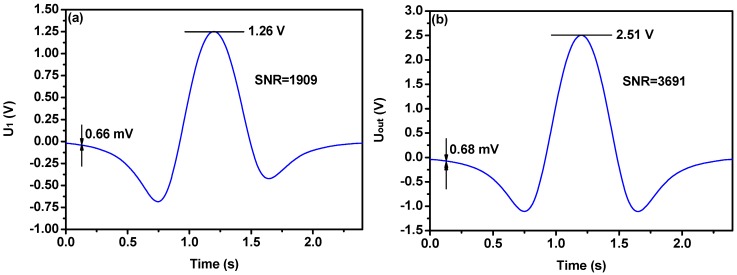
Experimental result. (**a**) The diagram of second harmonic signal when DFB-LD1 and lock-in amplifier 1 works alone, (**b**) The diagram of second harmonic signal in dual path lock-in system.

The water vapor sample with a concentration of 2000 ppmv was injected into the PAS cell. The second harmonic waveforms obtained in a single cycle as shown in Figure 4a,b. The second harmonic signal when the DFB-LD 1 and lock-in amplifier 1 worked alone, is shown in Figure 4a. The sensor noise was determined as a standard deviation from the signal far from the targeted absorption line. The signal and noise level is about 1.26 V and 0.66 mV for DFB-LD1. The corresponding SNR is about 1909. Figure 4b shows the second harmonic signal when the two DFB-LDs work simultaneously, the signal amplitude is about 2.51 V and the noise is about 0.68 mV. The SNR is improved to 3691. Compare one DFB-LD, the SNR increases 2 times. As can be seen from Figure 4, the dual path lock-in technique do not add additional noise. This shows that the wavelength modulation technology eliminated random interference noise such as optical noise. The main noise is the lock-in amplifier circuit noise.

### 4.3. Linearity and Long-Term Stability in Water Vapor Detection

**Figure 5:**
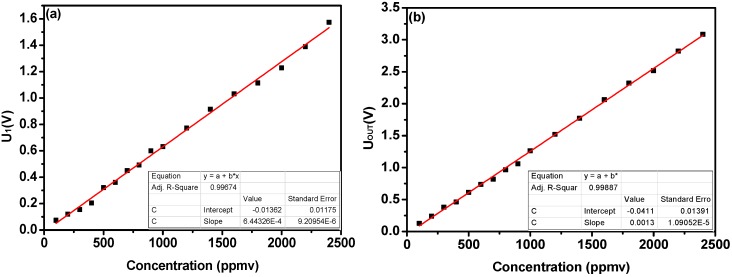
(**a**) Linearity test result with different water vapor concentration using DFB-LD 1 and lock-in amplifier 1, (**b**) Linearity test result with different water vapor concentration using dual path lock-in system.

In order to verify the linear response of the system, several water vapor samples of different concentrations from 100 ppmv to 2400 ppmv were generated by a dew-point generator. The linear response of the single DFB-LD 1 and lock-in amplifier 1 system is plotted in Figure 5a. The linearity is described by a linear equation with an R-square of 0.99674. Figure 5b depicts the linear response of the dual path lock-in system. A linear fit to the experimental data yields an R-square of 0.99887, indicating a good linear response of the sensor to H_2_O concentration.

**Figure 6:**
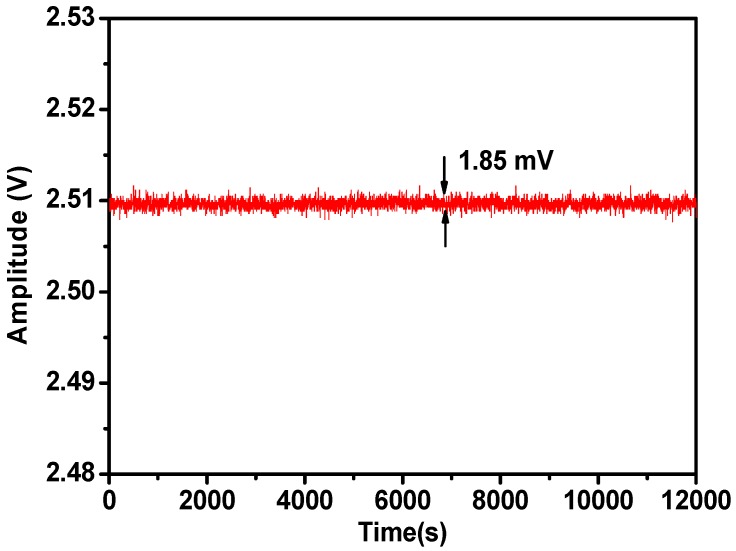
Long-term stability of dual path lock-in system, the water vapor concentration is 2000 ppmv.

Finally, to evaluate the long-term stability of the dual path lock-in system, the sensor system was used to measure the second harmonic signal amplitude of water vapor at 2000 ppmv. Measured second harmonic signal amplitude changes of water vapor over 3.3 h are displayed in Figure 6. It is observed that the second harmonic signal amplitude of water vapor was relatively stable during this period of time. The signal amplitude is about 2.509 V and the signal fluctuation is about 1.85 mV. The corresponding MDL is about 1.47 ppmv.

## 5. Conclusions

In the work reported here, we came up with a dual path lock-in technique to eliminate RAM and enhance SNR, which was verified by theoretical analysis and experiments. The results showed that a drastic reduction of RAM effect is possible using a subtraction circuit, based on a dual path lock-in. The published methods [8,11] used for RAM removal often caused signal amplitude reduction, but in our system, the signal amplitude could be increased two times by utilizing two DFB-LDs. Simultaneously, the noise analysis showed that it would not bring additional noise, so the SNR could be increased two times for water vapor detection and achieved a MDL of 1.47 ppmv. Finally, preliminary measurements of the dual path lock-in system response to water vapor concentrations have been made with pure N_2_ as a carrier gas, and the experimental results showed the relationship between second harmonic signal amplitude and the gas concentrations has a good linearity (R-square = 0.99887). The continuous monitoring of water vapor concentration levels for >3 h indicated the stability of the reported system. In practical applications, second harmonic waveforms can be used to measure parameters of probed gases. For example, they can be used to simultaneously measure temperature, pressure, velocity [20] and pressure broadening and pressure shift [21]. It is very simple and effective in practical applications. The dual path lock-in technique can be applied not only in the photoacoustic spectroscopy based on PAS cells, but also to quartz-enhanced photoacoustic spectroscopy [22] and cantilever-enhanced photoacoustic spectroscopy [23].
